# Inferring the age of breeders from easily measurable variables

**DOI:** 10.1038/s41598-022-19381-4

**Published:** 2022-09-23

**Authors:** Meritxell Genovart, Katarina Klementisová, Daniel Oro, Pol Fernández-López, Albert Bertolero, Frederic Bartumeus

**Affiliations:** 1 CEAB (CSIC), Carrer Accés Cala Sant Francesc, 14, 17300 Blanes, Catalonia, Spain; 2 IMEDEA (CSIC-UIB), Miquel Marquès 21, 07190 Esporles, Balearic Islands, Spain; 3 Associació Ornitològica Picampall de les Terres de l’Ebre, Amposta, Catalonia, Spain; 4 CREAF, Cerdanyola del Vallès, 08193 Barcelona, Catalonia, Spain; 5 ICREA, Passeig Lluis Companys 23, 08010 Barcelona, Catalonia, Spain

**Keywords:** Ecological modelling, Population dynamics

## Abstract

Age drives differences in fitness components typically due to lower performances of younger and senescent individuals, and changes in breeding age structure influence population dynamics and persistence. However, determining age and age structure is challenging in most species, where distinctive age features are lacking and available methods require substantial efforts or invasive procedures. Here we explore the potential to assess the age of breeders, or at least to identify young and senescent individuals, by measuring some breeding parameters partially driven by age (e.g. egg volume in birds). Taking advantage of a long-term population monitored seabird, we first assessed whether age influenced egg volume, and identified other factors driving this trait by using general linear models. Secondly, we developed and evaluated a machine learning algorithm to assess the age of breeders using measurable variables. We confirmed that both younger and older individuals performed worse (less and smaller eggs) than middle-aged individuals. Our ensemble training algorithm was only able to distinguish young individuals, but not senescent breeders. We propose to test the combined use of field monitoring, classic regression analysis and machine learning methods in other wild populations were measurable breeding parameters are partially driven by age, as a possible tool for assessing age structure in the wild.

## Introduction

Age is one of the most important factors affecting vital rates of individuals^1–4^. Age differences in traits influencing fitness, have been documented across a wide range of wild animals^1^; for instance, individuals typically improve their breeding performance over their first few breeding attempts and then it stabilizes before declining in old individuals due to senescence^2,5^. Age- or stage-specific vital rates are the fundamental components used to estimate population growth rates, understand population dynamics and assess the viability of populations^6–8^. Additionally, even if commonly neglected, age structure is usually variable in space and time, with important consequences on short term populations dynamics.

**Figure 1:**
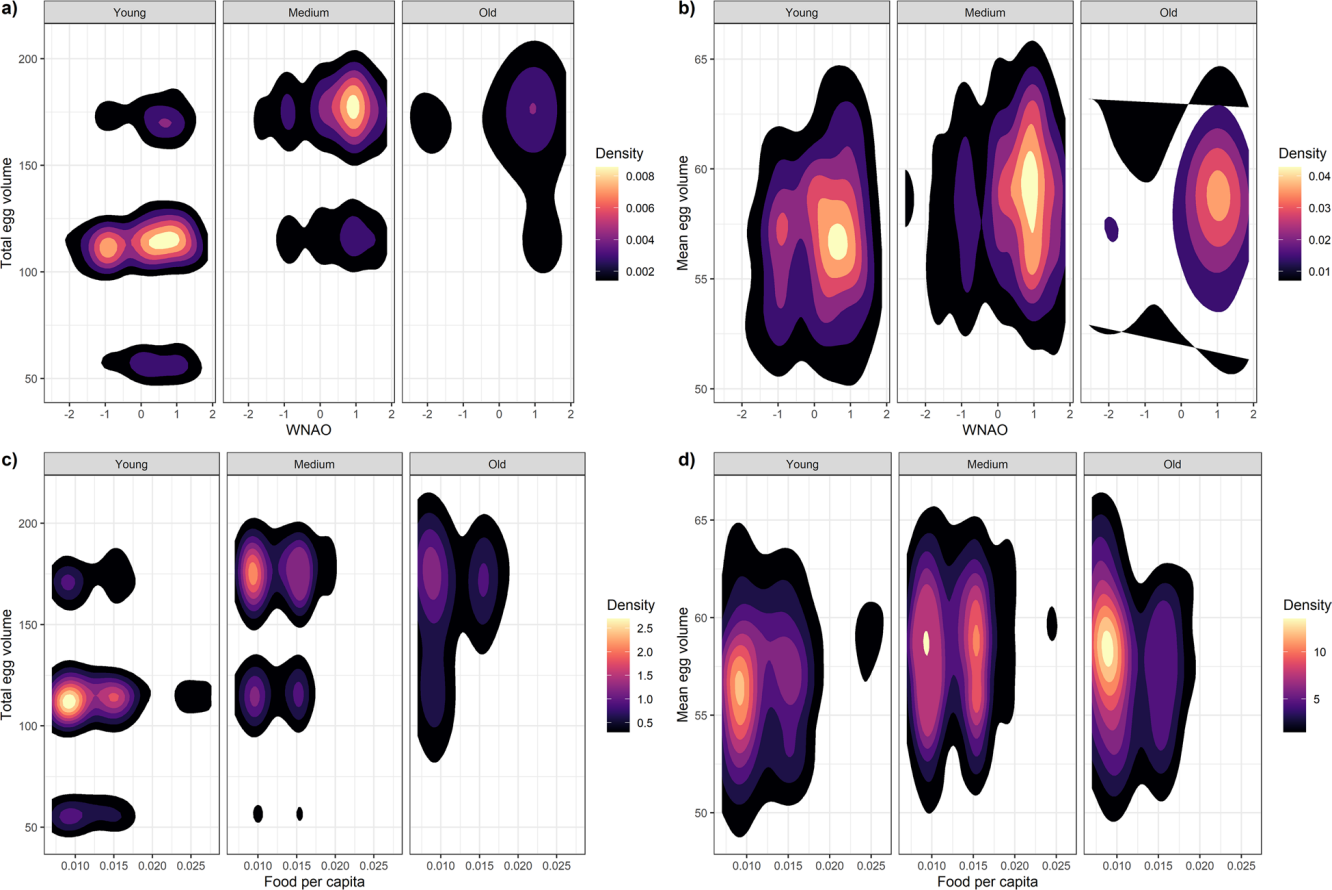
Density plots of the raw data depicting the effects of (**a**) age and winter climatic conditions (Winter NAO) on total egg volume, (**b**) age and winter climatic conditions (Winter NAO) on mean egg volume (**c**) age and food availability per capita on total egg volume and (**d**) age and food availability per capita on mean egg volume. Egg volume in cm$^{3}$. Young: 3–4 years old, middle-aged: 5–19 years old, and old individuals: > 20 years old.

**Figure 2:**
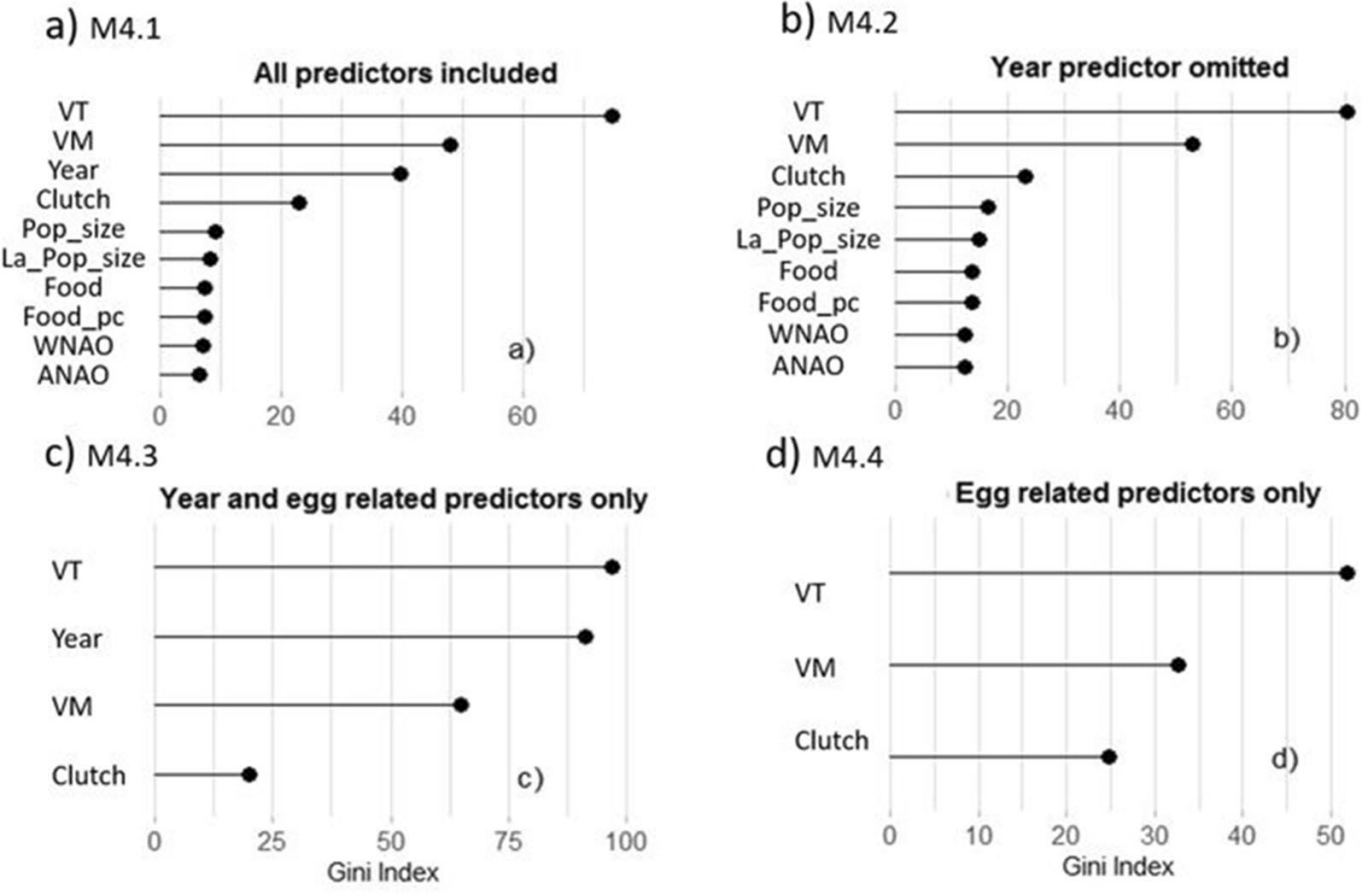
Relative importance of predictor variables (Gini Index) for the 2-age class model (3–4 years old/5+ year old gulls) on the four random forest classification models (M4; see “Methods”). *VT* total egg volume per nest, *VM* mean egg volume per nest, *Popsize* total population size of breeding pairs of Audouin’s and Yellow-legged gulls, *La*_*Popsize* population size of Audouin’s gull only, *Food* proxy of food availability, *Foodpc* proxy of per capita food availability, *WNAO* winter NAO Index, *ANAO* annual NAO Index. Values are aggregated from 3000 loop bootstrap of subsampling dataset used due to unbalanced age classes. Sample size: 2100 nests. Values of Gini Index are relative and comparable only within each figure part (**a**, **b**, **c** or **d**).

Variations in age structure may be due to disturbances or perturbations that are asymmetric across the life cycle or that affect the vital rates of the population^9^. Remarkably, these disturbances, and thus changes in age-structure, may be even more common under the actual human-caused global environmental change^10^. The importance of taking into account age structure may be especially relevant in long-lived species, where perturbations in the environment can alter age-structure for years before stable dynamics are recovered^11,12^. Estimation of age-structure may be also highly relevant in incipient populations or in invasive processes, where populations are likely far from equilibrium. Knowledge of age structure and its dynamics in populations is therefore crucial for a full understanding of the life history and ecological processes of organisms.

**Table 1:** Structure of the developed random forests models (M).

Age variable	Variable characteristics	Variance explained
M0:Continuous	3–28	1.9%
–	–	**Accuracy**
M1: 14 age classes	3, 4, 5, 6, 7, 8, 9, 10, 11, 12, 13, 14, 15, 16+	18%
M2: 6 age classes	3, 4, 5, 6, 7, 8–15, 16+	31%
M3: 3 age classes	3–4, 5–15, 16+	54%
M4: 2 age classes	3–4, 5+	71%

We first developed a regression random forest algorithm, considering age as a continuous variable (M0), and subsequent random forests took age as a factor variable to set four age-classes divisions (M1–M4).

**Table 2:** Averaged outputs of 2 age classes random forest analyses for 4 different versions of predictor combinations.

	All predictors	All predictors except Year	Year and egg related predictors	Only egg related predictors
	(M4.1)	(M4.2)	(M4.3)	(M4.4)
Accuracy (%)	71	71	70	70
Sensitivity (%)	74	74	69	76
Specificity (%)	69	67	71	64

Accuracy, sensitivity and specificity are model evaluation values. Parameters included in each model are: (1) Model M4.1, mean egg volume per nest (VM), total egg volume per nest (VT), clutch size (Clutch), year, fish landings (Food), fish landings available per capita (Foodpc), population size of Audouin’s gulls breeding pairs (LaPopsize), total population size of breeding pairs (Audouin’s gulls plus Yellow-legged gulls) (Popsize), winter North Atlantic Oscillation index (WNAO) and annual North Atlantic Oscillation index (ANAO), (2) Model M4.2, same as M4.1 without year factor, (3) Model M4.3, mean egg volume per nest, total egg volume per nest, clutch size and year and (4) Model M4.4, same as M4.3 without year factor.

Despite its importance, recording the age of breeding individuals and assessing breeding age structure in wild animal populations is challenging due to the common lack of external features indicating age. Although a few species have quantifiable physical changes as they become older, in most cases, external physical differences only allow to differentiate immature individuals from adults. These challenges limit our understanding of the dynamics of many wild animal species. In field studies, marking individuals when they are born allows population ecologists to monitor the age of individuals when resighted or recaptured and estimate important demographic parameters such as survival, recruitment or dispersal^13^. However, this method does not necessarily reflect the real breeding age structure, due to the variability in the proportion between marked /unmarked individuals each year and cohort effects, the difficulty to distinguishing between breeders and prospectors, and the potential for differential dispersal by age^14,15^. Numerous attempts have been carried out to reliably estimate the age of individuals in the wild, and molecular biomarkers have recently been the focus of an increasing number of studies^16–19^. Telomere length, DNA damage markers or DNA methylation (i.e. an epigenetic modification) are tools that open new exciting avenues of research, and as technological advances, it is likely that, especially DNA methylation, will become widely used in estimating population age structures^20^. However, these techniques require obtaining a blood sample of a sufficient number of individuals in the study population, which may represent a big challenge in the field.

Maternal age is known to affect breeding performance in many species^21–23^ and this may offer us the opportunity to assess the age of breeders in many taxa and study systems. In birds, as in other oviparous species, the egg contents reflect the age and condition of the mother at the time of egg laying; because developing embryos are completely dependent on these resources, egg size is positively related to nearly all offspring traits during all stages in their life cycle^24^. Moreover, egg size is in many cases, an easy to measure breeding performance trait so is a good candidate to help determine the age of individuals.

New quantitative methods are being developed for the analysis of ecological data. One example is random forests algorithms^25,26^, which are powerful statistical tools flexible enough to perform regression, classification, survival analysis, and unsupervised learning. Some key features make random forests suitable for ecological data analysis, such as high classification accuracy, the ability to model complex interactions among predictor variables, and the possibility to determine predictors’ importance and to incorporate missing values.

Here, we used random forest regression trees on data collected from a long-term monitoring (25-year) of colonial seabird in which egg characteristics are strongly affected by parental age^27^. Our aim was to develop and test a tool to estimate the age of breeding individuals, or at least to determine the proportion of young and senescent individuals in a population, by using easily measurable and observable variables in the field. We will also discuss the potential of using the same procedure to estimate the age of breeders and breeding age structure in other species and study populations.

## Results

We analysed data collected across 25-years (1994–2017, N = 2100 nests; 2018, N = 95 nests). Population size and environmental conditions showed large variability over the study period (Fig. S1).

### Factors driving mean and total egg volume

The age of breeders ranged from 3 (age of sexual maturity) to 28 years old (Fig. S2). Clutch size ranged from 1 to 5 eggs, and modal clutch size was 3 (60 % of the nests) (Fig. S3). Exploratory analyses of the data suggested that younger and older individuals performed worse (less and smaller eggs) (Figs. 1, S3, S4, S5).

When analysing data with a glm we observed that both mean and total egg volume in a clutch were best explained by the quadratic function of age (Tables S1–S2). When assessing other environmental factors explaining egg size variation, the best model showed that mean egg volume varied with clutch size, year and age in a quadratic manner (Tables S3, S4). Mean egg volume significantly increased with food availability (Model “food +#” vs “#”, Table S3). When looking at the AIC of the more complex models, ANAO (annual NAO) or WNAO (winter) indices ranked almost equally, but simpler models seemed to perform slightly better when including WNAO index instead of including ANAO, with larger eggs when larger WNAO index values (Fig. 1) (Model “WNAO” vs “ANAO”, Table S3). Regarding total egg volume in a clutch, the best model showed that total egg volume also varied with year and age in a quadratic manner (Tables S5, S6). Total egg volume significantly increased with food availability (Model “food +#” vs “#”, Table S6) and younger and older individuals showed poorer performances (Table S6; Fig. 1). The analysis showed that WNAO has more influence than ANAO when explaining total egg volume variation (Model “WNAO” vs “ANAO” and Model “WNAO +#” vs “ANAO +#”, Table S6), with larger eggs when winter NAO showed higher positive values (Fig. 1).

### Analytical tool to assess age from measurable variables

The total variance explained by the best random forest regression model (M0) was very low (1.9%) (Table 1). Based on that, and on the previous results analysing egg volume with the linear models, we decided to focus our analysis on random forest classification models and assess age classes instead of age as a continuous variable. The decrease in number of age classes resulted in an increase of the overall accuracy of the models’ classification (Table 1). We selected the model with only 2 age classes (3–4 years old and 5+ years old) as the model with enough accuracy to be used in further analyses (M4;71% accuracy). The four versions of the M4 algorithm taking different variables into account (M4.1–M4.4), showed similar accuracies (from 70 to 71%), but sensitivity and specificity seemed to slightly decrease and increase respectively when the year factor was considered (Tables 2, S7). All M4 model versions showed that total egg volume in a clutch is the most reliable predictor of age of breeders in the Audouin’s gull, followed by the mean egg volume per nest, except for version M4.3 where, once the climatic and environmental factors were excluded, year factor took more relevance (Fig. 2). Indeed, the factor ‘year’ was an important predictor in all model versions, whereas population sizes, NAO indices, and food were weak predictors (Fig. 2). When exploring the percentage of error for each year, we observed that there was great variability, with years with a percentage of error lower than 5% (e.g. 2008 and 2012) and other years with an error higher than 35% (e.g. 2003 or 2005) (Table S8). Also when exploring the percentage of error at different age classes we observed great variability, with really good accuracies at 3 and 4 years (6–8% of error) that sharply decrease with age (38% of error at 5 years old individuals) (Table S8).

### Additional testing on accuracy when predicting age

When using our tool to predict the age of breeders in 2018 at Barcelona harbour (N = 95 nests), we matched 76% and 73% of our age class guesses, with model versions M4.2 and M4.4 respectively. Differences from the proportion of breeders estimated by rings resighting (21%) and from our random forest tool (28%) were not statistically different (Fig. S6; $\tilde{\chi }^2=1.325, df=1, p<0.250$). The proportion of young breeders in 2018 in the seven colonies analysed varied from a maximum of 45% at the Salines Sant Antoni (SA) and a 43% at the Punta de la Banya (BN), to a non-detectable proportion of young breeders in Torrevieja or a 5% at Valencia harbour (Fig. S6). These differences in the proportion of young breeders among colonies were statistically significant and the accuracy of our tool was able to detect them (Fig. S6; $\tilde{\chi }^2=106.89, df=6, p<0.0001$).

We showed that the mean accuracy reached over 65% with relatively small sample sizes of the training dataset (N approximately 100 for both model versions) (Fig. S7).

## Discussion

We developed and evaluated a machine learning algorithm to assess the age of breeders by using measurable and easy-to-obtain variables. In our case study, Audouin’s gull populations, we confirmed that both younger and older individuals performed worse (less and smaller eggs) than middle-aged individuals. However, the developed analytical tool allowed us only to identify nests of young breeders, i.e, those individuals 3 and 4 years old, and was not able to identify reproductive senescence (i.e. lower breeding performance in the oldest individuals)^1,28^.

We showed that non-negligible differences in the proportion of young breeders may appear at the spatial scale between colonies of Audouin’s gull. Younger breeders in this species, as in many others, perform badly and have lower reproductive capacity, which is usually related to their lack of experience in acquiring sufficient quality and quantity of resources, such as food, mates, and territories^27,29,30^. Determining these young breeders proportions, and also those appearing at the temporal scale, may be highly relevant for understanding population dynamics and for guiding conservation actions of the study species. The global accuracy obtained with our algorithm for this species is not huge (71%), but we showed that this accuracy had been enough to compare observed proportions of young breeders.

Our algorithm was very reliable for identifying young breeders, although it failed ca 30% of the time when it classified some old individuals as young. The lack of accuracy and the incapability to detect reproductive senescence may be due, at least partially, to the small sample size of senescent individuals used to train the algorithm. This small sample size was probably due to both fewer senescent individuals marked in the population but also to fewer senescent breeders in years of hard environmental conditions^27^. Other factors limiting our tool, in this case misclassifying older than 4 years old as young, may be the existence of a strong individual heterogeneity in breeding capabilities not related to age, that are then translated in differences in egg-related metrics^31,32^. Individual variation is critical for the evolution of traits by the means of natural selection and exists within any population of living organisms^33,34^. However, we still have far to go before we reach a deep understanding of the causes and consequences of individual heterogeneity^33^. In our case study, perhaps identifying and understanding some individual information or traits related to individual heterogeneity (e.g cohort effects or egg coloration) and including them in the algorithm in the future, may help us to improve its accuracy. However, given the criticality and difficulty of assessing age in Audouin’s gull populations, we consider that the current approach offers a good balance between effort and benefits.

Assessing age of breeding individuals in wild populations is critical, and several approaches have been developed to solve this issue. Marking individuals when born allows researchers to monitor the age of individuals when resighted or recaptured, and even estimate important demographic parameters^13^. However, due to a possible unequal marking effort between years or breeding sites, resighting of marked individuals is in most cases not useful to estimate and compare age structures. Additionally, to assess breeding age structure you should also not only see the marked individual but also confirm its breeding status i.e. excluding prospectors. As technological advances broaden the scope of genomic analyses, it is likely that DNA methylation based age estimators will become widely used in determining age in wild populations^20,23^. However, nowadays epigenetic clocks still need to be developed across most species and individuals need to be captured and blood or tissue be sampled.

Maternal age is known to affect offspring performance in many species, with important ecological and evolutionary consequences^21,22,35^. This maternal age effect on breeding performance may offer us the opportunity to assess the age of breeders in many taxa and study systems through classification algorithms. The sole requirements for developing a classification algorithm for determining age are: (1) to have a monitored breeding population with some known aged individuals and (2) to identify measurable breeding parameters affected by maternal age. The way to determine the age of individuals and monitoring protocols could be very diverse and will depend on the species, the study system and the availability of human and logistic resources. This need for prior individual monitoring data of known age breeders for training the algorithm may be seen as a possible caveat of the usefulness of our approach. Even if individual monitoring it may be demanding in terms of resources, may generate scientific knowledge of high value in all major fields of biology^36,37^, and age-specific vital rates are the fundamental components used to estimate population growth rates and understand population dynamics. Therefore we advocate, when possible, for a monitoring program when evaluating population dynamics, especially in prospective population modelling (see also^38^). Additionally, as we showed, even if ours was a very large dataset, it seems that relatively small sample sizes would be enough for training the algorithm. The second requirement is the need to identify and measure breeding parameters affected by maternal age. There are many measurable characteristics affected by maternal age that could be used across taxa; for example, egg size, weight at birth, litter and offspring size or breeding success (this study^39–46^). Several examples would fit the two mentioned requirements. As in our case study, most colonial seabirds offer great opportunities to develop and apply this analytical tool, as many species allow marking many individuals at birth and monitor breeding performance. However, many other taxa can also meet these requirements, for example, birds and mammals breeding in nest boxes that can be manipulated with relative ease, or other species easy to capture and to monitor individually.

In our case study, the algorithm has been able to determine the age of a very small fraction of the population (individuals 3 and 4 years old) and with an accuracy of about 70%. Accuracy of classification algorithms will depend on species life history strategies and will increase with the strength of the maternal age effect. Additionally, caution should be taken when using trained algorithms based on other populations subject to particularly different natural selection pressures on life history traits. Another feature to take into account, is that this tool allows to assess age and age structure of the breeding population. In some cases, other complementary approximations will have to be carried out to obtain information about the non-breeding fraction of the population (i.e. immatures and individuals skipping reproduction). On the other side, forthcoming improved capacities for obtaining biometrics in the field, for example related with egg coloration or egg contents^47–50^, may be incorporated to improve power and accuracy of the algorithms.

## Conclusions

Our analytical tool failed to assessed the breeding age in our study species and only provided a way to estimate the proportion of young individuals in the breeding population. However, having an analytical tool to assess breeders’ age in wild populations may be a big step towards the understanding of population dynamics and for guiding biodiversity management. Thus we consider timely and relevant to incorporate any new tools and approaches that may allow us to estimate this critical demographic parameter to understand short-term population dynamics, and improve our long-term forecasting. The accuracy and usefulness of the approach will depend on the strength of the maternal age effect in the species and the ease with which monitoring can be carried out within the studied populations. Overall we propose the combined use of individual monitoring, classic regression analysis, and random forest methods, in other species to determine its utility to assess age and age structure in wild populations.

## Methods

### Study species

The Audouin’s gull *Larus audouinii* is a long-lived colonial seabird. The species is monogamous, exhibits age-related assortative mating, and is a bet-hedger, i.e. the species reduces the temporal variance in fitness at the expense of lowered arithmetic mean fitness^27^. Birds reach sexual maturity at 3 years old, annually lay one clutch with 1–5 eggs (modal value = 3), although few chicks survive to the fledgling age, except in good years when the strength of density-dependence is low^27^. Most recruitment to breeding population occurs when individuals reach sexual maturity or the year after, i.e. at 3–4 years old and it decreases sharply with age thereafter^51^. These 3–4 years old birds are young and inexperienced breeders, and would be named as “young” hereafters. Breeding colonies mostly occur in the western Mediterranean (80% of the total world population)^51^. Although individuals tend to show colony-site fidelity, dispersal between sites is common^52,53^, especially when sites are perturbed^15,54^. The species has been going through some drastic changes during last 40 years; first declared endangered around the 1980s, then “Least concern” due to a dramatic exponential growth in the Punta de la Banya colony and now in regression and again in the conservation spotlight^51^.

### Population monitoring

The long-term individual monitoring started in 1988 in the Punta de la Banya colony (40$^{\circ }$ 33$^{\prime }$ 36.5$^{\prime \prime }$ N 0$^{\circ }$ 39$^{\prime }$ 44.5$^{\prime \prime }$ E, Spain) and has continued until present day in this colony and at recently colonized sites (i.e. Sant Carles de la Ràpita, Tarragona port, and Barcelona port). Each breeding season, we marked a proportion of fledglings using alphanumeric plastic rings, which can be later resighted from the distance using spotting telescopes. Since 1994 to 2018, we also monitored nests of marked individuals i.e. of known age, until the clutch was completed, and measured the eggs. Audouin’s gulls, like many bird species, show assortative mating^27,55,56^, thus we assumed that the age of a non-marked partner was very close to that of the marked bird. We also measured eggs and calculated their volume in nests from which parental age was unknown. We calculated egg volume using the formula: Volume (cm$^{3}$) = 0.000476*length*width$^2$^57,58^. Finally, we counted the annual number of total nests as a proxy of population density^51^, and we did the same for Yellow-legged gulls (*L. michahellis*), the main competitor for food (see below).

### Environmental data

As in most age-related traits, egg volume and clutch size may also depend on environmental conditions changing each breeding season. To incorporate this variability, we annually recorded environmental data that may affect egg parameters in Audouin’s gulls. Fish trawling discards can represent over 70% of biomass of the diet during the breeding season^59^. Based on previous studies, we used the statistics of fish landings in April from the closest and largest harbor as a proxy for food availability when laying. We also calculated a per capita index of food availability (i.e. considering food density-dependence) by dividing fish landings by the number of Audouin’s and Yellow-legged gulls breeding in the study area. Food per capita values were standardized using z-transformation. Large-scale climatic indexes, such as the NAO (North Atlantic Oscillation), account for major variations in weather and climate around the world^60^. In the case at hand, the NAO index, especially during December–March (WNAO) is a proxy of environmental conditions affecting egg volume (authors unpublished data).

### Assessing factors driving egg volume

We analysed egg data from individuals of known age from 1994 to 2017. To develop a tool to infer parental age using egg volume in a clutch, we first assessed whether egg volume varied with the age of birds and identified the best age function or categorization explaining mean egg volume and total egg volume variation in a clutch. We tested age as a linear variable (Age) (to detect an improvement with age), age as a quadratic function (Age2) (to detect lower performance of young and old individuals) and the logarithmic function of age (LogAge) (to detect a lower performance only on young individuals); based on the graphical visualization of breeding parameters (Figs. S1–S5) we also used some age categorization; “Age14”, fourteen classes (ages from 3 to 15 were each treated as a separate class and all ages older than 16 were pooled in a single old class); “Age6”, six classes (ages from 3 to 6 were each treated as a separate class, ages from 7 to 15 were pooled a single Middle-aged class and ages older than 16 were pooled in a single Old class); “Age3”, three classes (ages 3 and 4 were pooled in a single Young class, ages from 5 to 15 were pooled in a Middle-aged class and ages older than 16 were pooled in an Old class); and “Age2”, two classes (ages 3 and 4 pooled as Young class and older than 5 pooled as Not Young). In each model we also included clutch size as a fixed factor, as it was previously showed to affect mean egg volume^57,59^. Then we used the best age function or structure to also identify the role of several environmental drivers. To this aim, we developed several general linear models, with mean egg volume as the dependent variable and several explanatory continuous covariates: annual North Atlantic Oscillation Index (ANAO), winter North Atlantic Oscillation index (WNAO), per capita food availability (Foodpc), as possible covariates, and year (Year) and clutch size (Clutch) as possible discrete fixed variables (i.e. factors). Clutch size was also included in each model. Only biologically meaningful models were tested, i.e. not all possible combinations of variables and their interactions were considered. The interactions between age and the annually changing variables (Year, Food, ANAO or WNAO) were also tested to explore whether gulls of different age react to annual changes differently. As the best age function explaining egg variation was the quadratic function (see “Results”), we also developed some models with the age structure that showed best accuracy at determining age with the Random Forest analysis (see below and results). The analyses were performed in R software version 4.1.2^61^ and RStudio^62^ using glm function from the stats package. Model selection was performed using Akaike Information Criterion (AIC).

### Analytical tool to assess age from measurable variables

We used random forests to predict the age of breeding birds using easily measurable and/or obtainable variables. We analysed egg data from individuals of known age from 1994 to 2017. As random forests can handle correlations among variables, we included all the parameters that could be playing a role: total egg volume per nest (sum of all egg volumes in a clutch) (VT), mean egg volume per nest (VM), ANAO index, WNAO index, population size of Audouin’s gulls (La_Popsize), total population size of gulls (Popsize), food availability (Food) and food availability per capita (Foodpc) as covariates, and clutch size (5 classes) and year (24 classes) as discrete explanatory variables (i.e. factors). We first used a random forest regression algorithm, considering age as a continuous variable^25^ (Table 1, see model M0). Based on observed differences (Figs. S3–S5) and given that the best age function explaining egg variability was the quadratic function (see “Results”), subsequent random forests took age as a factor variable (random forest classification models), and tried to differentiate groups with lower performance (young and old) from middle-aged individuals. As in the previous analyses, we established four different age class divisions or groupings (Table 1, models M1–M4). The different age structured models are defined as follows, M1 (“Age14”): fourteen classes—ages from 3 to 15 were each treated as a separate class and all ages of 16+ were pooled in a single class ‘Old’; M2 (“Age6”): six classes—ages from 3 to 6 were each treated as a separate class, ages from 7 to 15 were pooled a single class ‘Middle-aged’ and ages 16+ were pooled in a single class ‘Old’; M3 (“Age3”): three classes—ages 3 and 4 were pooled in a single class ‘Young’, ages from 5 to 15 were pooled in a class ‘Middle-aged’ and ages of 16+ were pooled in a class ‘Old’; and M4 (“Age2”): two classes—ages 3 and 4 pooled as class ‘Young’ and 5+ pooled as class ‘Others’. To avoid biases caused by different sample sizes of age classes (Fig. S2), subsets of data were sampled from the given class to create a balanced dataset for testing. A bootstrap (3000 iterations) was then applied to this subset to ensure the usage of all data available, as well as avoiding creation of biased subsets. Final results were attained by aggregating the results of all bootstraps. The best age structured model (M4, see Table 1 and results below) was then further expanded into four model versions including different variables: M4.1) all the variable predictors VT, VM, ANAO, WNAO, LaPopsize, Popsize, Food and Foodpc as covariates, and clutch and year factors; M4.2) as M4.1 but factor year omitted from the model; M4.3) factor year and egg measurement predictors (VT, VM and clutch) included; and M4.4) only egg measurement predictors included (VT, VM and clutch). These model versions were tested for assessing their accuracies and the applicability of the approach in case no environmental data was available (M4.3), when we had no information of the year in the training data set (M4.2) or only had data on egg characteristics (M4.4). Accuracy, sensitivity and specificity percentages were calculated for each different model. Accuracy was obtained by converting the out-of-bag error rate (OOB^63^ into an accuracy percentage (i.e., 100 * (1 − OOB)). Sensitivity measures the proportion (converted into percentages in our case) of positives correctly classified, and specificity measures the proportion of negatives correctly classified; positive and negative make reference here to the condition of belonging to a given age class. The Gini Index^63^ values for each of the predictors for each model were also obtained. These values are not comparable between different versions of the model, but within each version and they show the relative importance of each predictor. We further explored the performance of our algorithm by inspecting the data of misjudged nests. We evaluated the misclassified individuals according to model M4 (all predictors accounted). The model was trained several times in a bootstrap loop (3000 iterations) with different training sets. In each of these iterations, the resulting version of the model was used to predict the age class of each individual in the original dataset. In order to check which individuals were consistently misclassified across different training sets, we selected the individuals mismatched in at least half (i.e. 1500) of these models. We then estimate the percentage of error for each year and age class. Random Forest analysis was performed in R software^61^ version 4.1.2 (R Core Team 2017) in RStudio^62^ and using the randomForest package v.4.7-1.1^64^, the dplyr package v.1.0.9^65^ and caret package v.6.0-92^66^. R code script can be found in the Appendix in the Supporting information file.

### Additional testing on tool’s accuracy and applicability

To further evaluate the accuracy and applicability of this analytical tool, we used the trained random forest models to predict the age of breeders in one near colony, Barcelona’s harbour in 2018, a year that was not included to train the algorithm, and then used the results to compare spatial differences in age structure among colonies that year. To this purpose, we used model versions (M4.2) and (M4.4). We first compared the predicted proportion of young breeders estimated with our random forest tool with the one estimated from ring resightings of individuals of known age. We also estimated the proportion of young breeders at other colonies in 2018 based on ring resightings (Punta de la Banya, Castelló harbour, Salines Sant Antoni, Tarragona harbour, Torrevieja and Valencia harbour). For all colonies, we only considered resightings of those individuals showing breeding behaviour (i.e. alarm calls, incubating or observed with chicks) to avoid including prospectors or individuals non breeding. We then tested if there were differences among proportion of young breeders among colonies and if our accuracy was good enough for detecting these differences among colonies. Differences were tested with Chi square tests.

Our algorithm was based on a rich long-term monitoring database. To further assess the future applicability of the developed tool we also analysed changes in accuracy depending on the sample size of the training dataset. For this assessment we also used model versions (M4.2) and (M4.4).

## Supplementary Information

Supplementary Information.

## Data Availability

The datasets used and analysed during the current study are available from the corresponding author on reasonable request.
