# Supplementary material for: Inferring the age of breeders from easily measurable variables
Source: Sci Rep. 2022 Sep 23;12:15851. doi: 10.1038/s41598-022-19381-4 (PMC9508115; doi:10.1038/s41598-022-19381-4)
Supplement: Supplementary file 1 — Supplementary Information. [file 41598_2022_19381_MOESM1_ESM.docx]

**Supporting Information**

Inferring the age of breeders from easily measurable variables

Genovart M, Klementisová K, Oro D, Fernández-López P, Bertolero A, Bartumeus F

**Figure S1**. Population and environmental data for the study period (25 years of monitoring): a) changes in population size as number of breeding pairs; b) Standardized food per capita values, calculated using April statistical fish landings around the study colony and population sizes of Audouin’s and Yellow-legged gulls; c) Winter North Atlantic Oscillation (W_NAO_) index values.

**
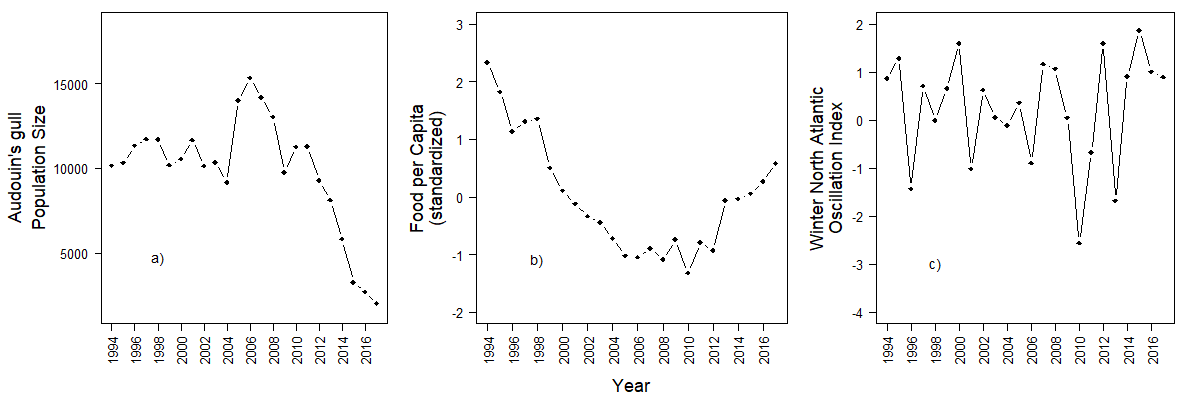
**

**Figure S2**. Frequencies of egg data for each age class in Audouin’s gull data set of known age from 1994-2017. Our dataset used was composed of 288 young individuals (3 and 4 years old) and 1812 ‘5+ years old gulls’ individuals (14% and 86%
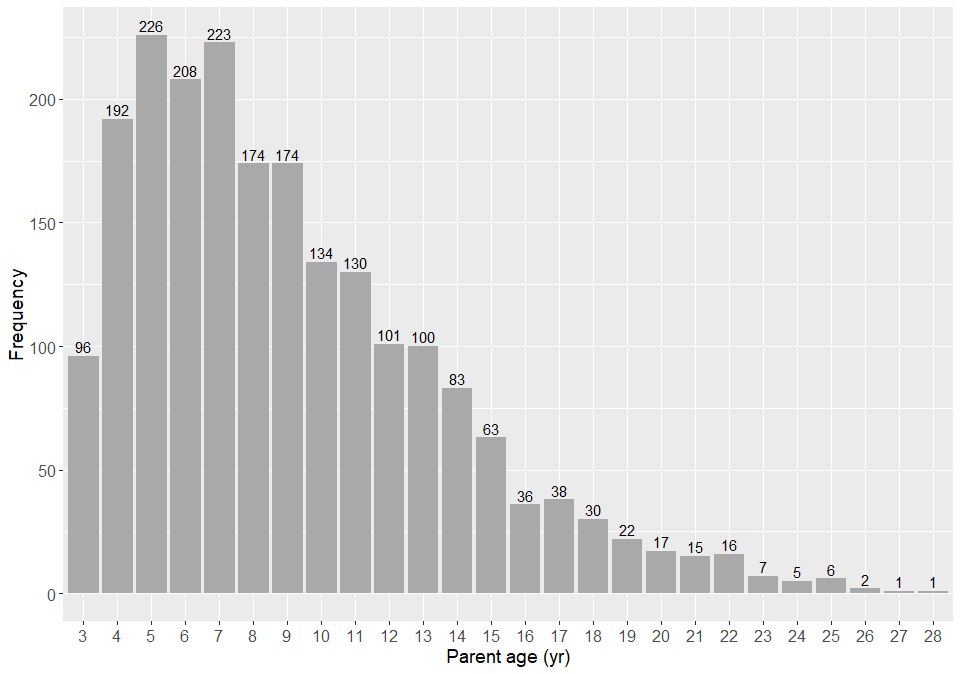
respectively).

**Figure S3**. Clutch size in Audouin’s gull of known age from 1994-2017. a) Frequencies of clutch size and b) mean clutch size by age.

**
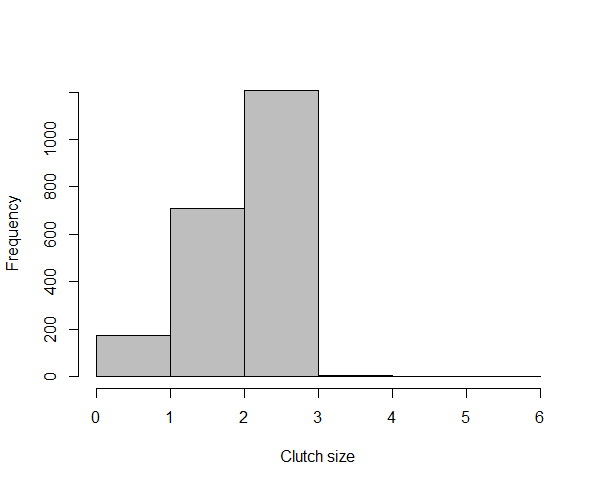
a)**

**
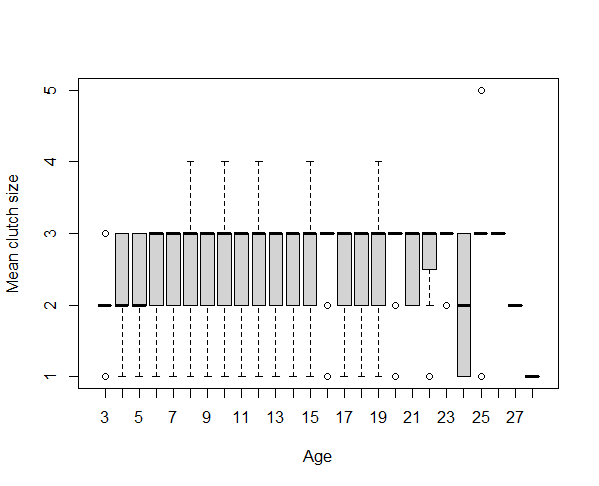
b)**

**Figure S4**. Violin plot showing summary statistics and the probability density function density (PDF) of mean egg volume (in cm3) by age. The black dot in the center of the box represents the median. The length of the box represents the interquartile range (IQR). The length of the line that extends out of the box represents the range. A wider PDF indicates that the value occurs more frequently, and a narrower density function indicates that the value occurs less frequently.

**
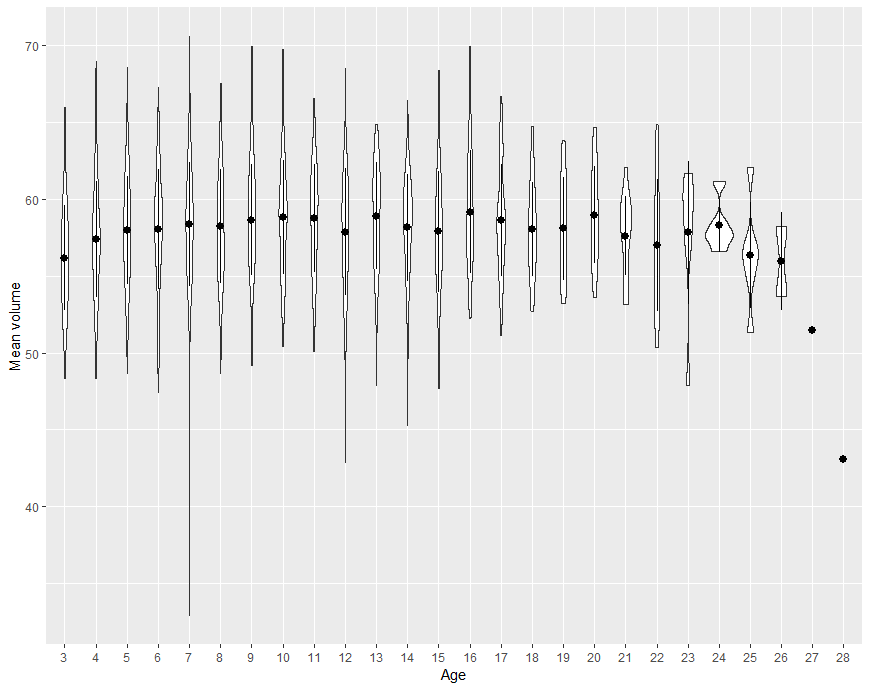
**

**Figure S5**. Violin plot showing summary statistics and the probability density function density (PDF) of total egg volume (in cm3) by age. The black dot in the center of the box represents the median. The length of the box represents the interquartile range (IQR). The length of the line that extends out of the box represents the range. A wider PDF indicates that the value occurs more frequently, and a narrower density function indicates that the value occurs less frequently. (in cm^3^) by age.

**
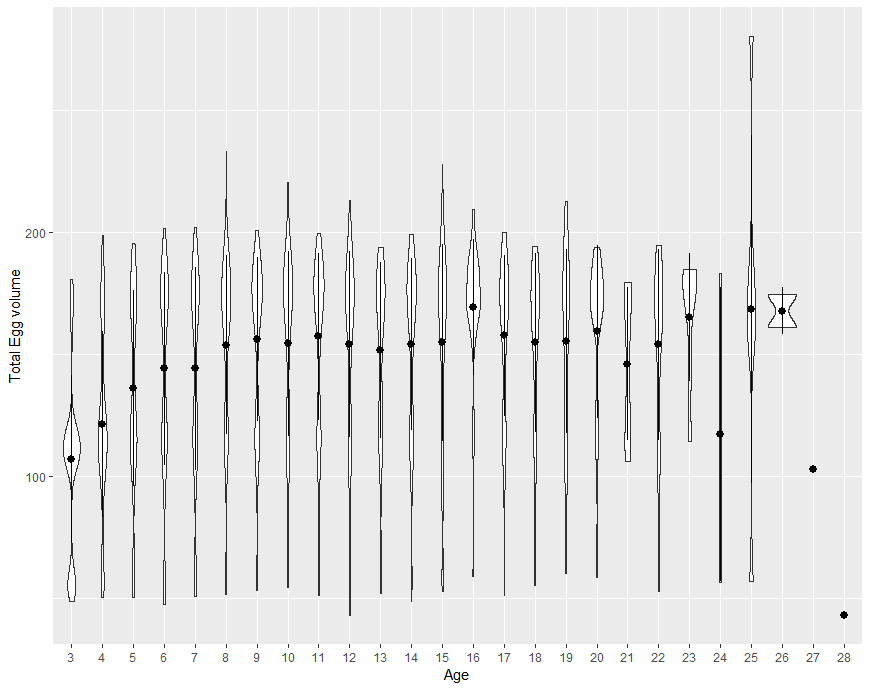
**

**Figure S6.** Proportion of young breeders in 2018 at different colonies (BCN: Barcelona’s harbour; BN: Punta de la Banya; CDT Castelló harbour; SA: Salines Sant Antoni; TGN: Tarragona harbour; TV: Torrevieja; VLC: Valencia harbour). First line (BCN_pred) show the proportion of young breeders at Barcelona harbour estimated with our Random Forests tool.

**
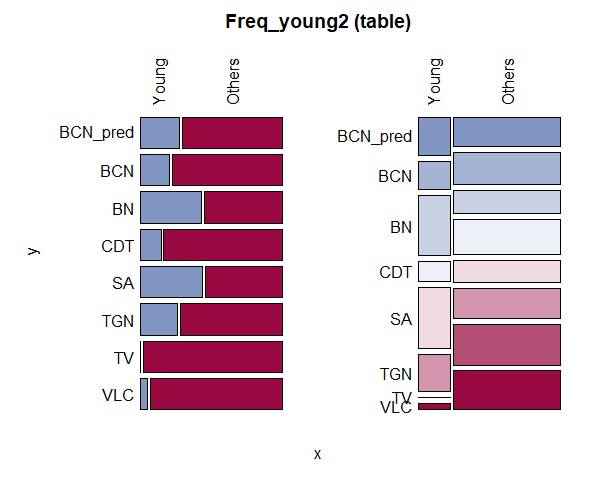
**

**Figure S7.** Percentage of accuracy depending on the size of the training data set. Analysis displayed for a) model version M4.2 including all variables except year and b) model version M4.4 including only egg related predictors. Sample size of nests = 2100.


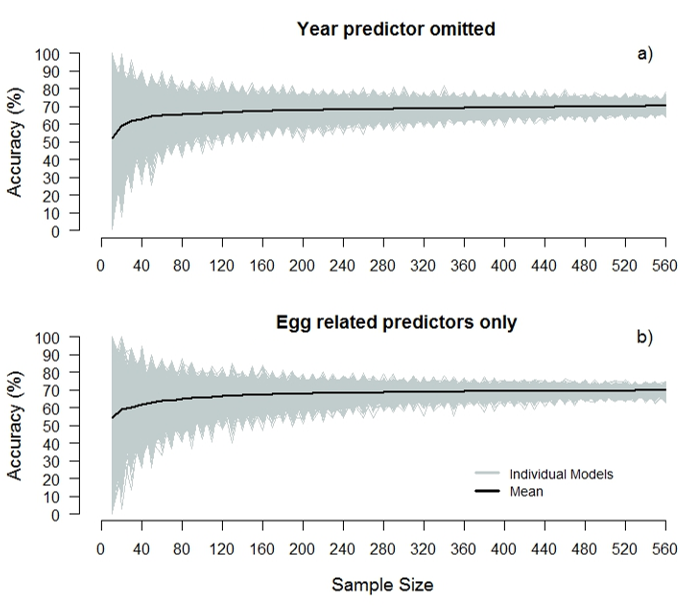


**Table S1:** Model selection table to determine the best age function or categorization explaining mean egg volume variation in a clutch. We test age as a linear variable (Age) (to detect an improvement with age), age as a quadratic function (Age2) (to detect lower performance of young and old individuals) and the logarithmic function of age (LogAge) (to detect a lower performance only on young individuals). We also use some age categorization;"Age14", fourteen classes (ages from 3 to 15 were each treated as a separate class and all ages older than 16 were pooled in a single old class); "Age6", six classes (ages from 3 to 6 were each treated as a separate class, ages from 7 to 15 were pooled a single Middle-aged class and ages older than 16 were pooled in a single Old class); "Age3", three classes (ages 3 and 4 were pooled in a single Young class, ages from 5 to 15 were pooled in a Middle-aged class and ages older than 16 were pooled in an Old class); and "Age2", two classes (ages 3 and 4 pooled as Young class and older than 5 pooled as Not Young). In each model we also included clutch size as a fixed factor, as it was previously showed to affect mean egg volume}. N = 2100.


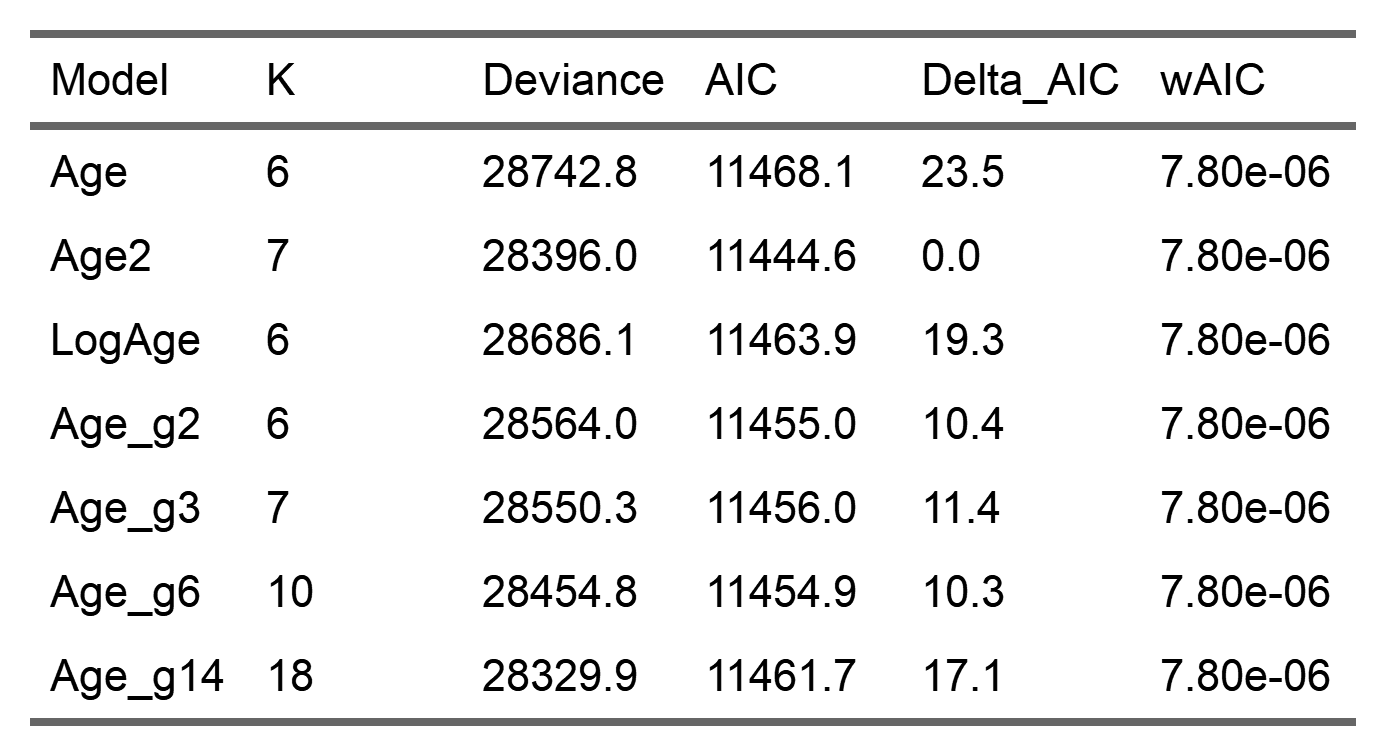


**Table S2:** Model selection table to determine the best age function or categorization explaining total egg volume variation in a clutch. We test age as a linear variable (Age) (to detect an improvement with age), age as a quadratic function (Age2) (to detect lower performance of young and old individuals) and the logarithmic function of age (LogAge) (to detect a lower performance only on young individuals). We also use some age categorization;"Age14", fourteen classes (ages from 3 to 15 were each treated as a separate class and all ages older than 16 were pooled in a single old class); "Age6", six classes (ages from 3 to 6 were each treated as a separate class, ages from 7 to 15 were pooled a single Middle-aged class and ages older than 16 were pooled in a single Old class); "Age3", three classes (ages 3 and 4 were pooled in a single Young class, ages from 5 to 15 were pooled in a Middle-aged class and ages older than 16 were pooled in an Old class); and "Age2", two classes (ages 3 and 4 pooled as Young class and older than 5 pooled as Not Young). In each model we also included clutch size as a fixed factor, as it was previously showed to affect mean egg volume}. N = 2100.


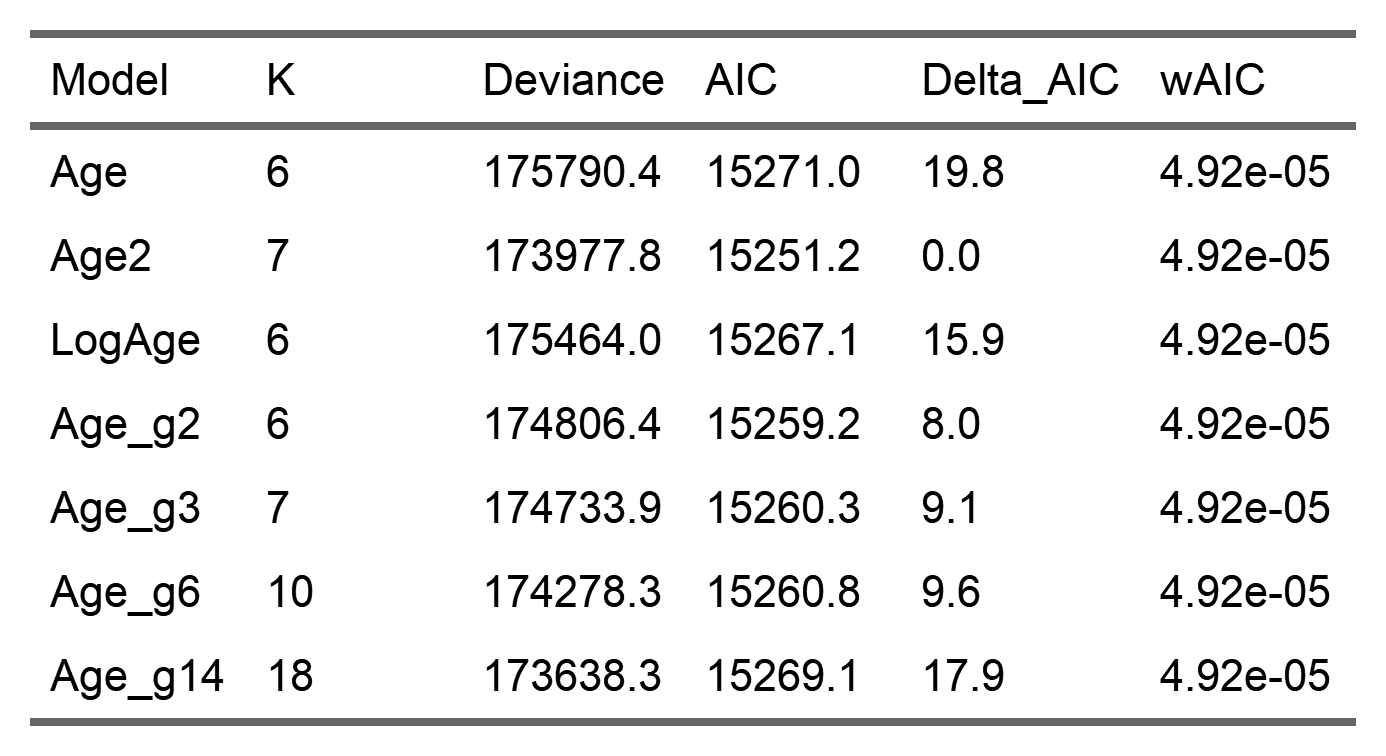


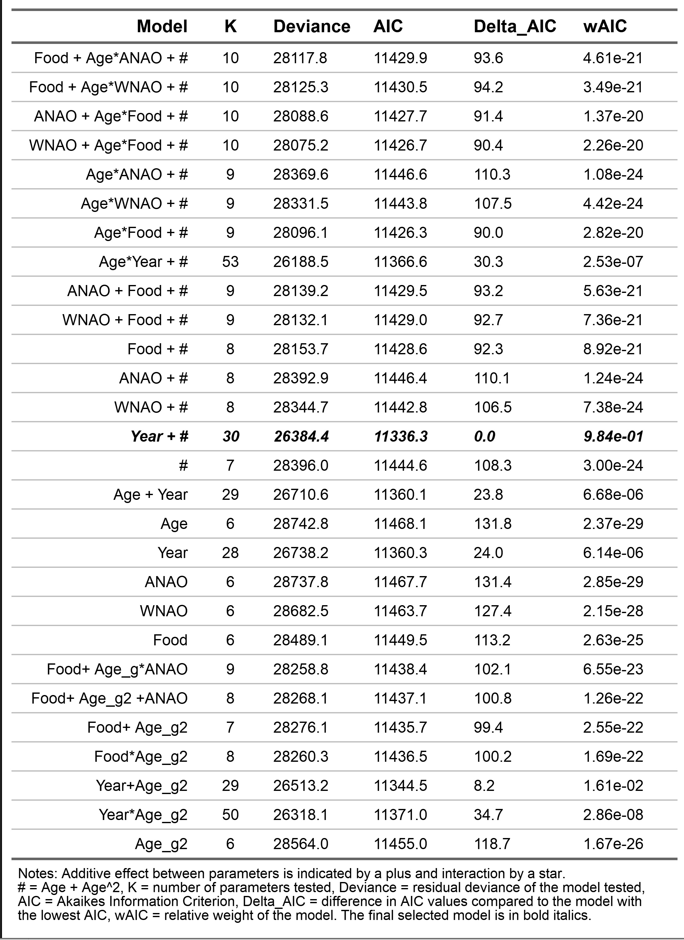
**Table S3:** Factors affecting mean egg volume in Audouin’s gull. We test the effects of Age, Annual/Winter North Atlantic Oscillation indices (ANAO and WNAO), food available per capita and global year conditions (Year) on the values of mean egg volume per nest of Audouin’s Gulls (*L. audouinii*). Each model included clutch size factor and models with ‘#’ include quadratic function of age. N = 2100.

**Table S4**: Summary of the mean egg analysis best model output. Model: Mean egg volume ~ Age + Age2 + Clutch size + Year. N = 2100. Parameters tested = 26.

| **Coefficient** | **Estimate** | **Std. Error** | **t value** | **Pr (>\|t\|)** |
| --- | --- | --- | --- | --- |
| Year1994/Clutch size 1 | 56.77 | 0.82 | 69.17 | < 2e-16 *** |
| Clutch size 2 | 0.54 | 0.30 | 1.77 | 0.08 . |
| Clutch size 3 | 1.21 | 0.30 | 3.99 | 6.73e-05 *** |
| Clutch size 4 | -1.62 | 1.63 | -1.00 | 0.32 |
| Clutch size 5 | 2.40 | 3.63 | 0.66 | 0.51 |
| Age | 0.38 | 0.07 | 5.27 | 1.53e-07 *** |
| Age^2^ | -0.02 | 0.003 | -5.06 | 4.60e-07 *** |
| Year1995 | 1.18 | 0.98 | 1.21 | 0.23 |
| Year1996 | 0.01 | 1.30 | 0.01 | 0.99 |
| Year1997 | -0.53 | 1.11 | -0.48 | 0.63 |
| Year1998 | -0.56 | 0.89 | -0.63 | 0.53 |
| Year1999 | -0.34 | 0.89 | -0.39 | 0.70 |
| Year2000 | -0.65 | 0.85 | -0.76 | 0.45 |
| Year2001 | -0.63 | 0.81 | -0.78 | 0.43 |
| Year2002 | -0.46 | 0.81 | -0.57 | 0.57 |
| Year2003 | -0.76 | 0.83 | -0.92 | 0.36 |
| Year2004 | -0.54 | 0.82 | -0.65 | 0.51 |
| Year2005 | -1.78 | 0.79 | -2.25 | 0.02 * |
| Year2006 | -2.63 | 0.80 | -3.29 | 0.001 ** |
| Year2007 | -1.39 | 0.80 | -1.74 | 0.08 . |
| Year2008 | -0.16 | 0.80 | -0.20 | 0.84 |
| Year2009 | -1.99 | 0.89 | -2.25 | 0.02 * |
| Year2010 | -1.22 | 0.82 | -1.49 | 0.14 |
| Year2011 | -1.70 | 0.84 | -2.03 | 0.04 * |
| Year2012 | -2.85 | 0.79 | -3.59 | 0.0003 *** |
| Year2013 | -3.05 | 0.79 | -3.84 | 0.0001 *** |
| Year2014 | -2.07 | 0.78 | -2.64 | 0.008 ** |
| Year2015 | -2.14 | 0.89 | -2.41 | 0.02 * |
| Year2016 | -0.92 | 0.89 | -1.03 | 0.30 |
| Year2017 | -0.58 | 0.86 | -0.68 | 0.50 |


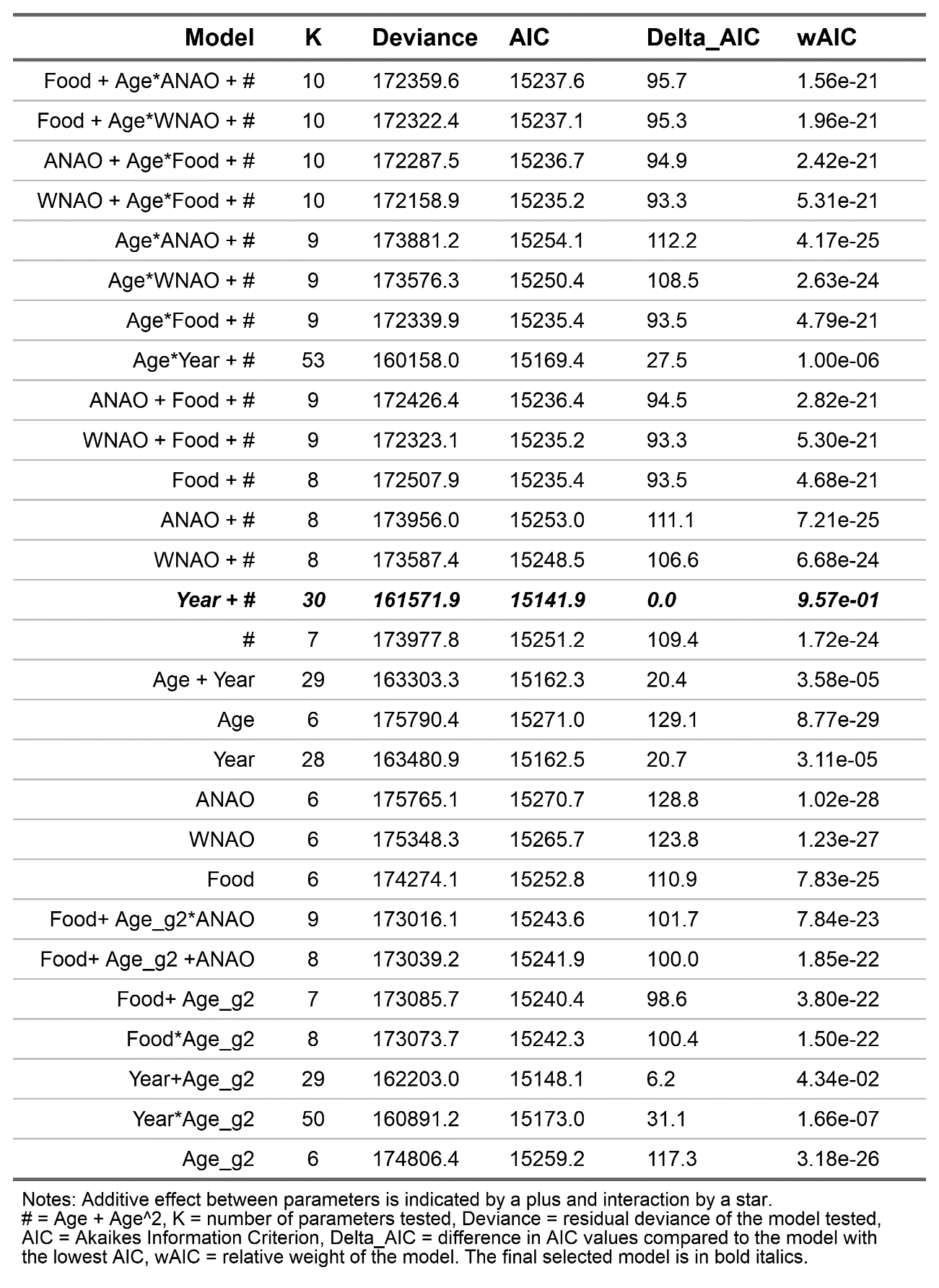
**Table S5:** Factors affecting total egg volume in Audouin’s gull. We test the effects of Age, Annual and Winter North Atlantic Oscillation indices (ANAO and WNAO), food available per capita and global year conditions (Year) on the values of total egg volume per nest of Audouin’s Gulls (*L. audouinii*). Each model included clutch size factor and models with ‘#’ include quadratic function of age. N = 2100.

**Table S6**: Summary of the total egg volume analysis best model output. The model assumes that total egg volume depends on age in a quadratic manner and year conditions, and clutch size is included as a factor effect. N = 2100.

| **Coefficient** | **Estimate** | **Std. Error** | **t value** | **Pr (>\|t\|)** |
| --- | --- | --- | --- | --- |
| Year1994/Clutch size 1 | 56.95 | 2.03 | 28.04 | < 2e-16 *** |
| Clutch size 2 | 57.83 | 0.75 | 76.74 | < 2e-16 *** |
| Clutch size 3 | 117.63 | 0.75 | 156.86 | < 2e-16 *** |
| Clutch size 4 | 164.44 | 4.04 | 40.73 | < 2e-16 *** |
| Clutch size 5 | 230.99 | 8.98 | 25.71 | < 2e-16 *** |
| Age | 0.88 | 0.18 | 4.94 | 8.48e-07 *** |
| Age^2^ | -0.03 | 0.01 | -4.71 | 2.64e-06 *** |
| Year1995 | 3.32 | 2.42 | 1.37 | 0.17 |
| Year1996 | 0.14 | 3.22 | 0.04 | 0.97 |
| Year1997 | -1.65 | 2.76 | -0.60 | 0.55 |
| Year1998 | -1.61 | -2.20 | 0.73 | 0.47 |
| Year1999 | -0.99 | 2.19 | -0.45 | 0.65 |
| Year2000 | -1.43 | 2.11 | -0.68 | 0.50 |
| Year2001 | -1.80 | 1.99 | - 0.90 | 0.37 |
| Year2002 | -1.04 | 1.99 | -0.52 | 0.60 |
| Year2003 | -2.15 | 2.04 | -1.05 | 0.29 |
| Year2004 | -1.243 | 2.03 | - 0.61 | 0.54 |
| Year2005 | -4.29 | 1.96 | -2.19 | 0.03 * |
| Year2006 | -6.39 | 1.98 | -3.23 | 0.001 ** |
| Year2007 | -3.69 | 1.98 | -1.87 | 0.06 . |
| Year2008 | -0.15 | 1.98 | -0.07 | 0.94 |
| Year2009 | -4.55 | 2.19 | -2.08 | 0.04 * |
| Year2010 | -3.30 | 2.03 | -1.63 | 0.10 |
| Year2011 | -4.60 | 2.07 | -2.22 | 0.03 * |
| Year2012 | -7.11 | 1.96 | -3.62 | 0.0003 *** |
| Year2013 | -7.503 | 1.97 | -3.816 | 0.0001 *** |
| Year2014 | -5.06 | 1.94 | - 2.611 | 0.009 ** |
| Year2015 | -5.42 | 2.19 | -2.472 | 0.01 * |
| Year2016 | -2.81 | 2.21 | -1.275 | 0.20 |
| Year2017 | -1.25 | 2.13 | -0.589 | 0.56 |

**Table S7**: Cross-testing values of the entire dataset (N = 2100) using all versions of the best model structure (M4) to further test the reliability of the random forest algorithm. Observed and predicted values are number of nests for young (3 and 4-year-old) and older gulls. Observed values come from the reading of the PVC rings of the breeders (see methods for more details).

|  | **Observed** | **Predicted** | | | | |
| --- | --- | --- | --- | --- | --- | --- |
|  |  | **M4.1** | **M4.2** | **M4.3** | **M4.4** |  |
| **Young** | 288 | 774 | 790 | 750 | 871 |  |
| **Others** | 1812 | 1326 | 1310 | 1638 | 1229 |  |
| **Matched predictions** | - | 1592  (76 %) | 1588  (76 %) | 1638  (78 %) | 1387  (66 %) |  |

**Table S8**. Percentage of error for each a) year and b) age classes. We show in bold those years with percentage of error lower than 5%. N indicates the sample size for this year or age class.

| **year** | **Error by year** | **N** |
| --- | --- | --- |
| 1994 | 71 | 24 |
| 1995 | 7 | 30 |
| 1996 | 27 | 11 |
| 1997 | 33 | 18 |
| 1998 | 46 | 50 |
| **1999** | **0** | **54** |
| 2000 | 22 | 68 |
| 2001 | 28 | 119 |
| 2002 | 23 | 119 |
| 2003 | 43 | 95 |
| 2004 | 19 | 99 |
| 2005 | 37 | 140 |
| 2006 | 34 | 125 |
| 2007 | 29 | 125 |
| **2008** | **4** | **127** |
| 2009 | 18 | 55 |
| 2010 | 39 | 102 |
| 2011 | 13 | 79 |
| **2012** | **4** | **158** |
| 2013 | 33 | 149 |
| 2014 | 29 | 184 |
| **2015** | **0** | **54** |
| 2016 | 38 | 50 |
| 2017 | 12 | 65 |

| **Age** | **Error by age** | **N** |
| --- | --- | --- |
| **3** | **0,06** | **96** |
| **4** | **0,08** | **192** |
| 5 | 0,38 | 226 |
| 6 | 0,32 | 208 |
| 7 | 0,29 | 223 |
| 8 | 0,22 | 174 |
| 9 | 0,29 | 174 |
| 10 | 0,27 | 134 |
| 11 | 0,18 | 130 |
| 12 | 0,30 | 101 |
| 13 | 0,27 | 100 |
| 14 | 0,22 | 83 |
| 15 | 0,27 | 63 |
| 16 | 0,17 | 36 |
| 17 | 0,34 | 38 |
| 18 | 0,23 | 30 |
| 19 | 0,32 | 22 |
| 20 | 0,06 | 17 |
| **21** | **0,00** | **15** |
| **22** | **0,06** | **16** |
| 23 | 0,29 | 7 |
| 24 | 0,20 | 5 |
| 25 | 0,33 | 6 |
| **26** | **0,00** | **2** |
| 27 | - | 0 |
| 28 | 1,00 | 1 |

a) b)

## Appendix

### R code: RF – Modelling

library(randomForest)

library(readr)

rf$Year<-as.factor(rf$Year)

rf$Clutch_size<-as.factor(rf$Clutch_size)

#######Age as covariate######################################

nboot=3000

actual<-rf$Edat

predicted<-c()

R2<-c()

#Loop

for(i in 1:nboot){

###model

model<-randomForest(Edat~., data=rf)

###outputs

predicted<-model$predicted

R2[i]<-1-(sum((actual-predicted)^2)/sum((actual-mean(actual))^2))

}

paste(100*range(R2))

paste(100*mean(R2))

########14 classes##########################################

rf14<-rf

rf14$Edat[rf14$Edat>=16]<- 16

table(rf14$Edat)

rf14$Edat<-as.factor(rf14$Edat)

#Loop

nboot=3000

acc14<-c()

for(i in 1:nboot){

###model

model<-randomForest(Edat~., data=rf14)

###get outputs

acc14[i]<-(100*(1-(mean(model$err.rate[,1]))))

}

paste(range(acc14))

paste(mean(acc14))

########6 classes##########################################

rf6<-rf

rf6$Edat[rf6$Edat>=7 & rf6$Edat<=15] <-10

rf6$Edat[rf6$Edat>=16] <-20

rf6$Edat<-as.factor(rf6$Edat)

levels(rf6$Edat)<-c("3", "4", "5", "6", "Medium", "Old")

table(rf6$Edat)

##BOOTSTRAP NEEDED FOR CLASS MEDIUM###

#Loop

nboot=3000

sub1<-rf6[rf6$Edat!="Medium",]

acc6<-c()

for(i in 1:nboot){

###balanced dataset for modelling

medium<-rf6[rf6$Edat=="Medium",]

m<-sample(nrow(medium),.15*nrow(medium))

train6<-rbind.data.frame(sub1, medium[m,])

###model

model<-randomForest(Edat~., data=train6)

###get outputs

acc6[i]<-(100*(1-(mean(model$err.rate[,1]))))

}

paste(range(acc6))

paste(mean(acc6))

########3 classes##########################################

rf3<-rf

rf3$Edat[rf3$Edat<=4] <-1

rf3$Edat[rf3$Edat>=5 & rf3$Edat<=15] <-2

rf3$Edat[rf3$Edat>=16] <-3

rf3$Edat<-as.factor(rf3$Edat)

levels(rf3$Edat)<-c("Young", "Medium", "Old")

table(rf3$Edat)

##BOOTSTRAP NEEDED FOR CLASS MEDIUM###

#Loop

nboot=3000

sub2<-rf3[rf3$Edat!="Medium",]

acc3<-c()

acc3_young<-c()

acc3_old<-c()

for(i in 1:nboot){

###balanced dataset for modelling

medium2<-rf3[rf3$Edat=="Medium",]

m2<-sample(nrow(medium2),.15*nrow(medium2))

train3<-rbind.data.frame(sub2, medium2[m2,])

###model

model<-randomForest(Edat~., data=train3)

###get outputs

acc3[i]<-(100*(1-(mean(model$err.rate[,1]))))

acc3_young[i]<-100*(1-model$confusion[1,4])

acc3_old[i]<-100*(1-model$confusion[3,4])

}

paste(range(acc3))

paste(mean(acc3))

#------------------------------#

paste(range(acc3_young))

paste(mean(acc3_young))

#------------------------------#

paste(range(acc3_old))

paste(mean(acc3_old))

########2 classes##########################################

rf2<-rf

rf2$Edat[rf2$Edat<=4] <-1

rf2$Edat[rf2$Edat>=5] <-2

rf2$Edat<-as.factor(rf2$Edat)

levels(rf2$Edat)<-c("Young", "Others")

table(rf2$Edat)

##BOOTSTRAP NEEDED FOR CLASS OTHERS###

#Loop

nboot=3000

sub3<-rf2[rf2$Edat=="Young",]

acc2<-c()

for(i in 1:nboot){

###balanced dataset for modelling

others<-rf2[rf2$Edat=="Others",]

o<-sample(nrow(others),.15*nrow(others))

train2<-rbind.data.frame(sub3, others[o,])

###model

model<-randomForest(Edat~., data=train2)

###get outputs

acc2[i]<-(100*(1-(mean(model$err.rate[,1]))))

}

paste(range(acc2))

paste(mean(acc2))

MODELLING ONLY USING NESTS WITH 3 EGGS

library(randomForest)

library(readr)

rf$Year<-as.factor(rf$Year)

rf<-rf[rf$Mida_posta==3,]

#######Age as covariate######################################

actual<-rf$Edat

###model

model<-randomForest(Edat~., data=rf)

###outputs

predicted<-model$predicted

R2<-1-(sum((actual-predicted)^2)/sum((actual-mean(actual))^2))

paste(100*(R2))

########14 classes##########################################

rf14<-rf

rf14$Edat[rf14$Edat>=16]<- 16

table(rf14$Edat)

rf14$Edat<-as.factor(rf14$Edat)

#Loop

nboot=3000

acc14<-c()

for(i in 1:nboot){

###model

model<-randomForest(Edat~., data=rf14)

###get outputs

acc14[i]<-(100*(1-(mean(model$err.rate[,1]))))

}

paste(range(acc14))

paste(mean(acc14))

########6 classes##########################################

rf6<-rf

rf6$Edat[rf6$Edat>=7 & rf6$Edat<=15] <-10

rf6$Edat[rf6$Edat>=16] <-20

rf6$Edat<-as.factor(rf6$Edat)

levels(rf6$Edat)<-c("3", "4", "5", "6", "Medium", "Old")

table(rf6$Edat)

##BOOTSTRAP NEEDED FOR CLASS MEDIUM###

#Loop

nboot=3000

sub1<-rf6[rf6$Edat!="Medium",]

acc6<-c()

for(i in 1:nboot){

###balanced dataset for modelling

medium<-rf6[rf6$Edat=="Medium",]

m<-sample(nrow(medium),.15*nrow(medium))

train6<-rbind.data.frame(sub1, medium[m,])

###model

model<-randomForest(Edat~., data=train6)

###get outputs

acc6[i]<-(100*(1-(mean(model$err.rate[,1]))))

}

paste(range(acc6))

paste(mean(acc6))

########3 classes##########################################

rf3<-rf

rf3$Edat[rf3$Edat<=4] <-1

rf3$Edat[rf3$Edat>=5 & rf3$Edat<=15] <-2

rf3$Edat[rf3$Edat>=16] <-3

rf3$Edat<-as.factor(rf3$Edat)

levels(rf3$Edat)<-c("Young", "Medium", "Old")

table(rf3$Edat)

##BOOTSTRAP NEEDED FOR CLASS MEDIUM###

#Loop

nboot=3000

sub2<-rf3[rf3$Edat!="Medium",]

acc3<-c()

acc3_young<-c()

acc3_old<-c()

for(i in 1:nboot){

###balanced dataset for modelling

medium2<-rf3[rf3$Edat=="Medium",]

m2<-sample(nrow(medium2),.1*nrow(medium2))

train3<-rbind.data.frame(sub2, medium2[m2,])

###model

model<-randomForest(Edat~., data=train3)

###get outputs

acc3[i]<-(100*(1-(mean(model$err.rate[,1]))))

acc3_young[i]<-100*(1-model$confusion[1,4])

acc3_old[i]<-100*(1-model$confusion[3,4])

}

paste(range(acc3))

paste(mean(acc3))

#------------------------------#

paste(range(acc3_young))

paste(mean(acc3_young))

#------------------------------#

paste(range(acc3_old))

paste(mean(acc3_old))

########2 classes##########################################

rf2<-rf

rf2$Edat[rf2$Edat<=15] <-1

rf2$Edat[rf2$Edat>=16] <-2

rf2$Edat<-as.factor(rf2$Edat)

levels(rf2$Edat)<-c("Others", "Old")

table(rf2$Edat)

##BOOTSTRAP NEEDED FOR CLASS OTHERS###

#Loop

nboot=30

sub3<-rf2[rf2$Edat=="Old",]

acc2<-c()

acc2_old<-c()

for(i in 1:nboot){

###balanced dataset for modelling

others<-rf2[rf2$Edat=="Others",]

o<-sample(nrow(others),.15*nrow(others))

train2<-rbind.data.frame(sub3, others[o,])

###model

model<-randomForest(Edat~., data=train2)

###get outputs

acc2[i]<-(100*(1-(mean(model$err.rate[,1]))))

acc2_old[i]<-(100*(1-model$confusion[2,3]))

}

paste(range(acc2))

paste(mean(acc2))

paste(range(acc2_old))

paste(mean(acc2_old))

### R code: RF – 2 age class analysis

library(randomForest)

library(caret)

library(readr)

library(dplyr)

random1$Clutch_size<-as.factor(random1$Clutch_size)

random1$Year<-as.factor(random1$Year)

random1$Edat[random1$Edat<=4]<-1

random1$Edat[random1$Edat>=5]<-2

random1$Edat<-as.factor(random1$Edat)

levels(random1$Edat)<-c("Young", "Others")

rf<-random1

table(rf$Edat)

y2011$Colony[1:1091]<-"Banya"

y2011$Colony[1092:1166]<-"Rapita"

y2011$Colony<-as.factor(y2011$Colony)

y2011$Clutch_size<-as.factor(y2011$Clutch_size)

y2011$Year<-as.factor(y2011$Year<-2011)

levels(y2011$Year) <- levels(rf$Year)

y2011$Year[y2011$Year=="1994"]<-"2011"

levels(y2011$Clutch_size) <- levels(rf$Clutch_size)

table(y2011$Colony)

#----------------------------ALL VARIABLES-------------------------------------#

###Loop

nboot=3000

all_y2011_pred<-matrix(nrow = (nrow(y2011)), ncol = nboot)

varImp<-matrix(nrow = (ncol(rf)-1), ncol = nboot)

confM<-list()

acc<-c()

sens<-c()

spec<-c()

all_check<-matrix(nrow = (nrow(rf)), ncol = nboot)

for(i in 1:nboot){

###balanced subset of all data

sub<-rf[rf$Edat=="Young",]

o<-rf[rf$Edat=="Others",]

others<-sample(nrow(o),.15*nrow(o))

train<-rbind.data.frame(sub, o[others,])

###model with train subset

model<-randomForest(Edat~., data=train)

###get outputs

all_y2011_pred[,i]<-predict(model, y2011)

varImp[,i]<-importance(model, class=Edat, scale=T)

confM[[i]]<-(confusionMatrix(data=model$predicted, reference = train$Edat))

acc[i]<-100*(confM[[i]]$overall[1])

sens[i]<-confM[[i]]$byClass[1]

spec[i]<-confM[[i]]$byClass[2]

all_check[,i]<-predict(model, rf)

}

#####outputs

print(range(acc))

outputs<-cbind.data.frame(acc, sens, spec)

all_2cl<-as.data.frame(apply(outputs, 2, mean))

rownames(varImp)<-rownames(importance(model, class=Edat, scale=T))

all_GiniTable<-as.data.frame(apply(varImp, 1, mean))

colnames(all_GiniTable)="Gini_Index"

dimnames(all_check)[[2]]<-sapply(1:nboot, function(i) paste("M", i, sep="_"))

all_check_2cl<-all_check

###predictions of 2011 file

dimnames(all_y2011_pred)[[2]]<-sapply(1:nboot, function(i) paste("RMod",i, sep="_"))

all_y2011_pred<-as.data.frame(apply(all_y2011_pred, 1, mean))

all_y2011_pred<-round(all_y2011_pred, digits=0)

colnames(all_y2011_pred)[1]="Pred_Age"

all_y2011_pred$Pred_Age<-as.factor(all_y2011_pred$Pred_Age)

levels(all_y2011_pred$Pred_Age)<-c("Young", "Others")

all_y2011_2cl<-bind_cols(y2011, all_y2011_pred)

#----------------------------YEAR OMITTED------------------------------------#

rf1<-random1[,-5]

###Loop

nboot=3000

noyear_y2011_pred<-matrix(nrow = (nrow(y2011)), ncol = nboot)

noyear_varImp<-matrix(nrow = (ncol(rf1)-1), ncol = nboot)

noyear_confM<-list()

noyear_acc<-c()

noyear_sens<-c()

noyear_spec<-c()

noyear_check<-matrix(nrow = (nrow(rf1)), ncol = nboot)

for(i in 1:nboot){

###balanced subset of all data

sub1<-rf1[rf1$Edat=="Young",]

o1<-rf1[rf1$Edat=="Others",]

others1<-sample(nrow(o1),.15*nrow(o1))

train1<-rbind.data.frame(sub1, o1[others1,])

###model with train subset

model<-randomForest(Edat~., data=train1)

###get outputs

noyear_y2011_pred[,i]<-predict(model, y2011)

noyear_varImp[,i]<-importance(model, class=Edat, scale=T)

noyear_confM[[i]]<-(confusionMatrix(data=model$predicted, reference = train1$Edat))

noyear_acc[i]<-100*(noyear_confM[[i]]$overall[1])

noyear_sens[i]<-noyear_confM[[i]]$byClass[1]

noyear_spec[i]<-noyear_confM[[i]]$byClass[2]

noyear_check[,i]<-predict(model, rf1)

}

#####outputs

print(range(noyear_acc))

noyear_outputs<-cbind.data.frame(noyear_acc, noyear_sens, noyear_spec)

noyear_2cl<-as.data.frame(apply(noyear_outputs, 2, mean))

rownames(noyear_varImp)<-rownames(importance(model, class=Edat, scale=T))

noyear_GiniTable<-as.data.frame(apply(noyear_varImp, 1, mean))

colnames(noyear_GiniTable)="Gini_Index"

dimnames(noyear_check)[[2]]<-sapply(1:nboot, function(i) paste("M", i, sep="_"))

noyear_check_2cl<-noyear_check

###predictions of 2011 file

dimnames(noyear_y2011_pred)[[2]]<-sapply(1:nboot, function(i) paste("RMod",i, sep="_"))

noyear_y2011_pred<-as.data.frame(apply(noyear_y2011_pred, 1, mean))

noyear_y2011_pred<-round(noyear_y2011_pred, digits=0)

colnames(noyear_y2011_pred)[1]="Pred_Age"

noyear_y2011_pred$Pred_Age<-as.factor(noyear_y2011_pred$Pred_Age)

levels(noyear_y2011_pred$Pred_Age)<-c("Young", "Others")

noyear_y2011_2cl<-bind_cols(y2011, noyear_y2011_pred)

#----------------------EGG MEASUREMENTS AND YEAR ONLY--------------------------#

rf2<-random1[,c(1:5)]

###Loop

nboot=3000

yearegg_y2011_pred<-matrix(nrow = (nrow(y2011)), ncol = nboot)

yearegg_varImp<-matrix(nrow = (ncol(rf2)-1), ncol = nboot)

yearegg_confM<-list()

yearegg_acc<-c()

yearegg_sens<-c()

yearegg_spec<-c()

yearegg_check<-matrix(nrow = (nrow(rf2)), ncol = nboot)

for(i in 1:nboot){

###balanced subset of all data

sub2<-rf2[rf2$Edat=="Young",]

o2<-rf2[rf2$Edat=="Others",]

others2<-sample(nrow(o2),.15*nrow(o2))

train2<-rbind.data.frame(sub2, o2[others2,])

###model with train subset

model<-randomForest(Edat~., data=train2)

###get outputs

yearegg_y2011_pred[,i]<-predict(model, y2011)

yearegg_varImp[,i]<-importance(model, class=Edat, scale=T)

yearegg_confM[[i]]<-(confusionMatrix(data=model$predicted, reference = train2$Edat))

yearegg_acc[i]<-100*(yearegg_confM[[i]]$overall[1])

yearegg_sens[i]<-yearegg_confM[[i]]$byClass[1]

yearegg_spec[i]<-yearegg_confM[[i]]$byClass[2]

yearegg_check[,i]<-predict(model, rf2)

}

#####outputs

print(range(yearegg_acc))

yearegg_outputs<-cbind.data.frame(yearegg_acc, yearegg_sens, yearegg_spec)

yearegg_2cl<-as.data.frame(apply(yearegg_outputs, 2, mean))

rownames(yearegg_varImp)<-rownames(importance(model, class=Edat, scale=T))

yearegg_GiniTable<-as.data.frame(apply(yearegg_varImp, 1, mean))

colnames(yearegg_GiniTable)="Gini_Index"

dimnames(yearegg_check)[[2]]<-sapply(1:nboot, function(i) paste("M", i, sep="_"))

yearegg_check_2cl<-yearegg_check

###predictions of 2011 file

dimnames(yearegg_y2011_pred)[[2]]<-sapply(1:nboot, function(i) paste("RMod",i, sep="_"))

yearegg_y2011_pred<-as.data.frame(apply(yearegg_y2011_pred, 1, mean))

yearegg_y2011_pred<-round(yearegg_y2011_pred, digits=0)

colnames(yearegg_y2011_pred)[1]="Pred_Age"

yearegg_y2011_pred$Pred_Age<-as.factor(yearegg_y2011_pred$Pred_Age)

levels(yearegg_y2011_pred$Pred_Age)<-c("Young", "Others")

yearegg_y2011_2cl<-bind_cols(y2011, yearegg_y2011_pred)

#---------------------------EGG MEASUREMENTS ONLY----------------------------#

rf3<-random1[,c(1:4)]

###Loop

nboot=3000

eggonly_y2011_pred<-matrix(nrow = (nrow(y2011)), ncol = nboot)

eggonly_varImp<-matrix(nrow = (ncol(rf3)-1), ncol = nboot)

eggonly_confM<-list()

eggonly_acc<-c()

eggonly_sens<-c()

eggonly_spec<-c()

eggonly_check<-matrix(nrow = (nrow(rf3)), ncol = nboot)

for(i in 1:nboot){

###balanced subset of all data

sub3<-rf3[rf3$Edat=="Young",]

o3<-rf3[rf3$Edat=="Others",]

others3<-sample(nrow(o3),.15*nrow(o3))

train3<-rbind.data.frame(sub3, o3[others3,])

###model with train subset

model<-randomForest(Edat~., data=train3)

###get outputs

eggonly_y2011_pred[,i]<-predict(model, y2011)

eggonly_varImp[,i]<-importance(model, class=Edat, scale=T)

eggonly_confM[[i]]<-(confusionMatrix(data=model$predicted, reference = train3$Edat))

eggonly_acc[i]<-100*(eggonly_confM[[i]]$overall[1])

eggonly_sens[i]<-eggonly_confM[[i]]$byClass[1]

eggonly_spec[i]<-eggonly_confM[[i]]$byClass[2]

eggonly_check[,i]<-predict(model, rf3)

}

#####outputs

print(range(eggonly_acc))

eggonly_outputs<-cbind.data.frame(eggonly_acc, eggonly_sens, eggonly_spec)

eggonly_2cl<-as.data.frame(apply(eggonly_outputs, 2, mean))

rownames(eggonly_varImp)<-rownames(importance(model, class=Edat, scale=T))

eggonly_GiniTable<-as.data.frame(apply(eggonly_varImp, 1, mean))

colnames(eggonly_GiniTable)="Gini_Index"

dimnames(eggonly_check)[[2]]<-sapply(1:nboot, function(i) paste("M", i, sep="_"))

eggonly_check_2cl<-eggonly_check

###predictions of 2011 file

dimnames(eggonly_y2011_pred)[[2]]<-sapply(1:nboot, function(i) paste("RMod",i, sep="_"))

eggonly_y2011_pred<-as.data.frame(apply(eggonly_y2011_pred, 1, mean))

eggonly_y2011_pred<-round(eggonly_y2011_pred, digits=0)

colnames(eggonly_y2011_pred)[1]="Pred_Age"

eggonly_y2011_pred$Pred_Age<-as.factor(eggonly_y2011_pred$Pred_Age)

levels(eggonly_y2011_pred$Pred_Age)<-c("Young", "Others")

eggonly_y2011_2cl<-bind_cols(y2011, eggonly_y2011_pred)

y2018<-y2018[,-c(9,12)]

y2018$Clutch_size<-as.factor(y2018$Clutch_size)

levels(y2018$Clutch_size) <- levels(random1$Clutch_size)

y2018$Edat[y2018$Edat<=4]<-1

y2018$Edat[y2018$Edat>=5]<-2

y2018$Edat<-as.factor(y2018$Edat)

levels(y2018$Edat)<-c("Young", "Others")

print(ori<-table(y2018$Edat))

prop.table(ori) * 100

#----------------------------YEAR OMITTED-------------------------------------#

rf1<-rf

###Loop

nboot=3000

noyear_y2018_pred<-matrix(nrow = (nrow(y2018)), ncol = nboot)

for(i in 1:nboot){

###balanced subset of all data

sub1<-rf1[rf1$Edat=="Young",]

o1<-rf1[rf1$Edat=="Others",]

others1<-sample(nrow(o1),.15*nrow(o1))

train1<-rbind.data.frame(sub1, o1[others1,])

###model with train subset

model<-randomForest(Edat~., data=train1)

###get outputs

noyear_y2018_pred[,i]<-predict(model, y2018)

}

#####outputs

noyear_y2018_pred<-as.data.frame(apply(noyear_y2018_pred, 1, mean))

noyear_y2018_pred<-round(noyear_y2018_pred, digits=0)

colnames(noyear_y2018_pred)[1]="Pred_Age"

noyear_y2018_pred$Pred_Age<-as.factor(noyear_y2018_pred$Pred_Age)

levels(noyear_y2018_pred$Pred_Age)<-c("Young", "Others")

noyear_y2018_2cl<-bind_cols(y2018, noyear_y2018_pred)

confusionMatrix(data=noyear_y2018_2cl$Pred_Age, reference = noyear_y2018_2cl$Edat)

all.equal.character(noyear_y2018_2cl$Edat, noyear_y2018_2cl$Pred_Age)

#---------------------------------EGG ONLY-------------------------------------#

rf3<-rf[,c(1:4)]

###Loop

nboot=3000

eggonly_y2018_pred<-matrix(nrow = (nrow(y2018)), ncol = nboot)

for(i in 1:nboot){

###balanced subset of all data

sub3<-rf3[rf3$Edat=="Young",]

o3<-rf3[rf3$Edat=="Others",]

others3<-sample(nrow(o3),.15*nrow(o3))

train3<-rbind.data.frame(sub3, o3[others3,])

###model with train subset

model<-randomForest(Edat~., data=train3)

###get outputs

eggonly_y2018_pred[,i]<-predict(model, y2018)

}

#####outputs

eggonly_y2018_pred<-as.data.frame(apply(eggonly_y2018_pred, 1, mean))

eggonly_y2018_pred<-round(eggonly_y2018_pred, digits=0)

colnames(eggonly_y2018_pred)[1]="Pred_Age"

eggonly_y2018_pred$Pred_Age<-as.factor(eggonly_y2018_pred$Pred_Age)

levels(eggonly_y2018_pred$Pred_Age)<-c("Young", "Others")

eggonly_y2018_2cl<-bind_cols(y2018, eggonly_y2018_pred)

confusionMatrix(data=eggonly_y2018_2cl$Pred_Age, reference = eggonly_y2018_2cl$Edat)

all.equal.character(eggonly_y2018_2cl$Edat, eggonly_y2018_2cl$Pred_Age)

### R code: RF – Accuracy versus sample size analysis

library(randomForest)

library(caret)

library(dplyr)

library(readr)

rf$Edat[rf$Edat<=4]<-1

rf$Edat[rf$Edat>=5]<-2

rf$Edat<-as.factor(rf$Edat)

levels(rf$Edat)<-c("Young", "Others")

rf$Clutch_size<-as.factor(rf$Clutch_size)

rf<-rf[,-5]

table(rf$Edat)

#####

nboot=3000

N=seq(5,280,5)

results_accss<-matrix(ncol = length(N), nrow=nboot)

for(n in 1:nboot){

v<-c()

for(i in N){

s1<-sample(which(rf$Edat == "Young"), i)

s2<-sample(which(rf$Edat == "Others"), i)

rf1<-rf[c(s1,s2),]

model<-randomForest(Edat~., data=rf1)

v[which(N==i)]<- 100*(1-mean(model$err.rate[,1]))

}

results_accss[n,]<-v

}

results_accss<-as.data.frame(results_accss)

ave_results_accss<-as.data.frame(apply(results_accss, 2, mean))

ave_results_accss$N<-2*N

VERSION 4

rf<-rf[,-c(5:11)]

table(rf$Edat)

#####

nboot=3000

N=seq(5,280,5)

results_accss_eggonly<-matrix(ncol = length(N), nrow=nboot)

for(n in 1:nboot){

v<-c()

for(i in N){

s1<-sample(which(rf$Edat == "Young"), i)

s2<-sample(which(rf$Edat == "Others"), i)

rf1<-rf[c(s1,s2),]

model<-randomForest(Edat~., data=rf1)

v[which(N==i)]<- 100*(1-mean(model$err.rate[,1]))

}

results_accss_eggonly[n,]<-v

}

results_accss_eggonly<-as.data.frame(results_accss_eggonly)

ave_results_accss_eggonly<-as.data.frame(apply(results_accss_eggonly, 2, mean))

ave_results_accss_eggonly$N<-2*N
